# Sarcoidosis in the setting of combination ipilimumab and nivolumab immunotherapy: a case report & review of the literature

**DOI:** 10.1186/s40425-016-0199-9

**Published:** 2016-12-20

**Authors:** Joshua E. Reuss, Paul R. Kunk, Anne M. Stowman, Alejandro A. Gru, Craig L. Slingluff, Elizabeth M. Gaughan

**Affiliations:** 1 Department of Medicine, Division of Hematology-Oncology, University of Virginia Health System, PO Box 800716, Charlottesville, 22908 VA USA; 2 Department of Pathology, Division of Anatomic Pathology, University of Virginia Health System, PO Box 800716, Charlottesville, 22908 VA USA; 3 Department of Surgery, Division of Surgical Oncology, University of Virginia Health System, PO Box 800716, Charlottesville, 22908 VA USA

**Keywords:** Metastatic melanoma, Sarcoidosis, Checkpoint inhibitor, Ipilimumab, Nivolumab, Immune-related adverse event, Combination immunotherapy

## Abstract

**Background:**

We report a case of sarcoidosis in a patient with metastatic melanoma managed with combination ipilimumab/nivolumab. Sarcoid development has been linked with single agent immunotherapy but, to our knowledge, it has not been reported with combination ipilimumab/nivolumab treatment. This case raises unique management challenges for both the melanoma and the immunotherapy-related toxicity.

**Case presentation:**

A 46 year old Caucasian female with M1c-metastatic melanoma was managed with ipilimumab/nivolumab combination. Patient experienced response in baseline lesions but developed new clinical and radiographic findings. Biopsy of new lesions at two different sites both demonstrated tumefactive sarcoidosis. Staining of the biopsy tissue for PD-L1 expression demonstrated strong PD-L1 staining of the histiocytes and lymphocytes within the granulomas. Monotherapy nivolumab was continued without progression of sarcoid findings or clinical deterioration.

**Conclusions:**

Tissue biopsy for evaluation of new lesions on immunotherapy is an important step to help guide decision making, as non-melanoma lesions can mimic disease progression.

## Background

The treatment options for metastatic melanoma have improved dramatically with the advent of novel immunotherapies, specifically checkpoint inhibitors. Such agents include ipilimumab, a fully humanized monoclonal antibody against cytotoxic T-lymphocyte-associated antigen-4 (CTLA-4), as well as nivolumab and pembrolizumab, IgG4 anti-programmed-death 1 (anti-PD1) immune checkpoint inhibitor antibodies [1–4]. Prior to the approval of these agents, FDA-approved therapies for melanoma were limited by toxicity and low efficacy.

Currently, initial therapy for many newly-diagnosed patients with advanced melanoma is either anti-PD1 monotherapy or the combination of ipilimumab and nivolumab [3, 4]. The novel mechanisms of action of checkpoint inhibitors have resulted in a unique set of side effects, so called immune-related adverse events (irAEs). These toxicities can involve any organ system, though most commonly skin, gastrointestinal tract, and the endocrine system [1–4]. While less frequent, interstitial lung disease and pneumonitis have been documented [2, 3]. Sarcoidosis and sarcoid-like granulomatosis have been reported in melanoma patients treated with single agent ipilimumab and single-agent anti-PD1 therapy (Table 1) [5–14]. Sarcoid development has also been reported in sarcoma patients managed with pembrolizumab [15]. Given that development of sarcoidosis can mimic disease progression, accurate identification of this process is imperative. We report the first known case, to our knowledge, of tumefactive sarcoidosis following combination treatment with ipilimumab and nivolumab in a patient with metastatic melanoma, highlighting the importance of tissue diagnosis and raising management challenges.

**Table 1:** Cases of immunotherapy induced sarcoid and sarcoid-like granulomatous reaction in metastatic melanoma

Age*, Sex	Stage	Immunotherapy (cycle prior to sarcoid diagnosis)	Melanoma Response	Sarcoid Organ Involvement	Symptoms related to Sarcoid	Sarcoid Treatment Required	Reference
67 F	IV	Ipilimumab (4+)	stable disease	lung, mediastinum, skin	dyspnea	unknown	[5]
62 F	IV	Ipilimumab (not reported)	stable disease	mediastinal LN, skin, bronchus	none	No	[6]
49 M	IV	Ipilimumab (4)	complete response	mediastinal/hilar LN	none	N0	[7]
48 F	IV	Ipilimumab (2)	progression	Widespread LN, lung, spleen	dyspnea, cough, fatigue	Yes - steroids	[8]
63 M	IV	Ipilimumab (4)	progression	lung	dyspnea, dry cough, hypoxia	Yes-steroids	[9]
57 M	IIIB	Ipilimumab (6)	Unknown	lung, hilar LN, skin	none	No	[10]
55 M	IIIB	Ipilimumab (2)	progression	lung, mediastinal/hilar LN, skin	dyspnea, hypoxia	Yes-steroids	[11]
37 M	IV	Ipilimumab (4)	stable disease	mediastinal/hilar LN, brain	fatigue, arthralgia, anorexia, weight loss, headache	Yes-steroids	[12]
44 M	IV	Ipilimumab (4)	partial response	spleen	none	No	[13]
57 M	IV	Nivolumab (not reported)	complete response	mediastinal/hilar LN, skin	none	No	[14]

*Age at initial diagnosis of melanoma

## Case presentation

**Fig. 1:**
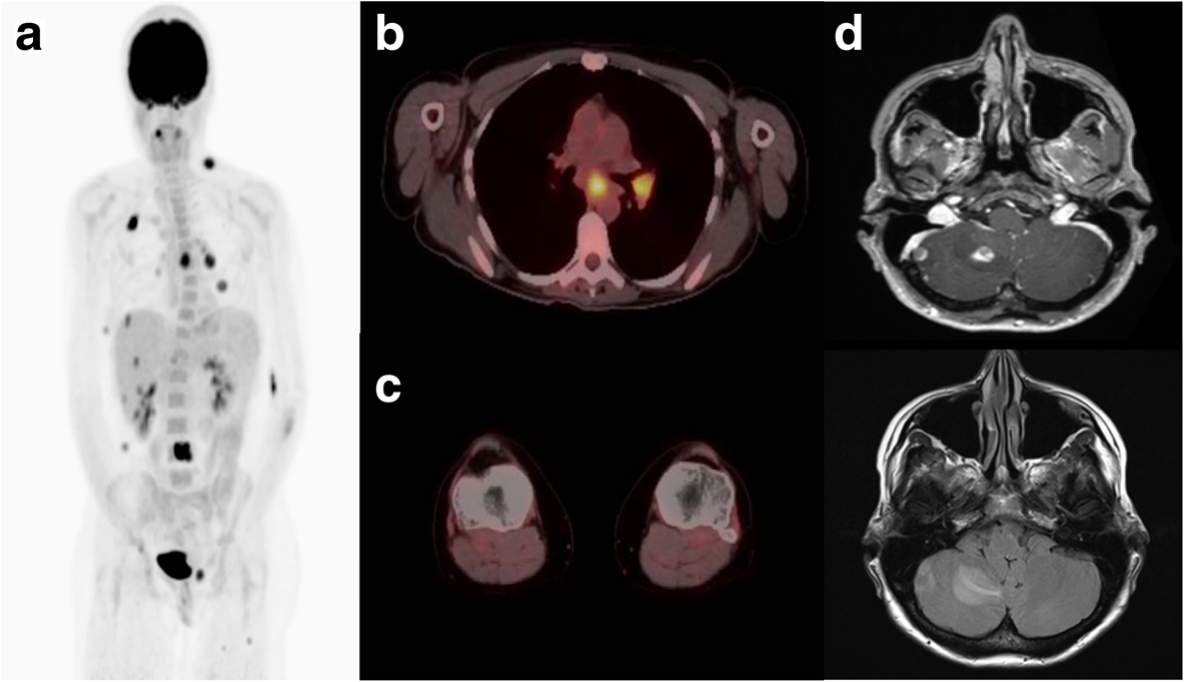
Imaging at Time of Diagnosis Before Treatment. **a** Scout PET scan showing cervical/mediastinal/inguinal adenopathy and vertebral lesions. **b** Cross section of mediastinum with increased update in mediastinal nodes (SUV_max_ 10 in subcarinal node). **c** Cross section of knees with no increased uptake. **d** MRI brain with two right cerebellar metastases with vasogenic edema

**Fig. 2:**
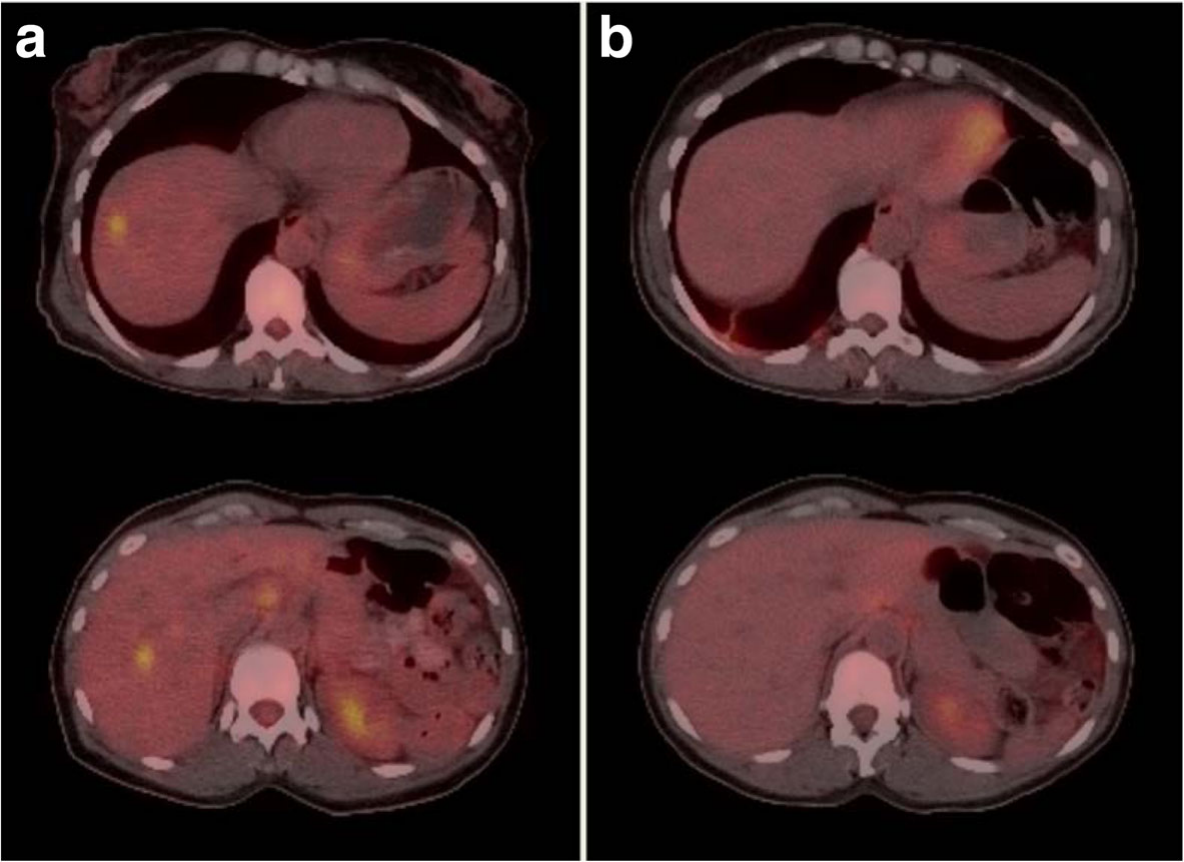
PET-CT of Hepatic Metastases Before and After Combination Immunotherapy. **a** PET-CT scan at times of diagnosis of with several hypermetabolic hepatic lesions consistent with metastases. **b** PET-CT scan after 4 cycles of nivolumab plus ipilimumab and 3 cycles of nivolumab maintenance

A 32-year-old female was diagnosed with pT3bN0M0, stage IIB, melanoma of the left anterior distal thigh in 2001. She was managed with wide excision, and sentinel lymph node evaluation was negative. The patient was observed and remained free of disease until 2015 when she presented to her dermatologist with subcutaneous lesions in the left groin and upper back. Punch biopsy of a left inguinal nodule demonstrated *BRAF* wild-type malignant melanoma. Diagnostic imaging with PET/CT and brain MRI demonstrated brain metastases, pulmonary nodules, left hilar and subcarinal lymphadenopathy, liver metastases, and several bone and soft tissue lesions (Figs. 1 and 2). Brain lesions were managed with Gamma Knife radiosurgery with good tolerance.

She commenced combination immunotherapy treatment with ipilimumab 3 mg/kg and nivolumab 1 mg/kg every 3 weeks. After the four combination doses, imaging demonstrated improvement in soft tissue disease, pulmonary and liver lesions, as well as brain metastases. The baseline subcarinal and left hilar adenopathy did not change in size. Treatment was well tolerated with grade 1 diarrhea, nausea, and rash, as well as the development of hypothyroidism requiring supplementation. She was transitioned to maintenance nivolumab 3 mg/kg every 2 weeks.

**Fig. 3:**
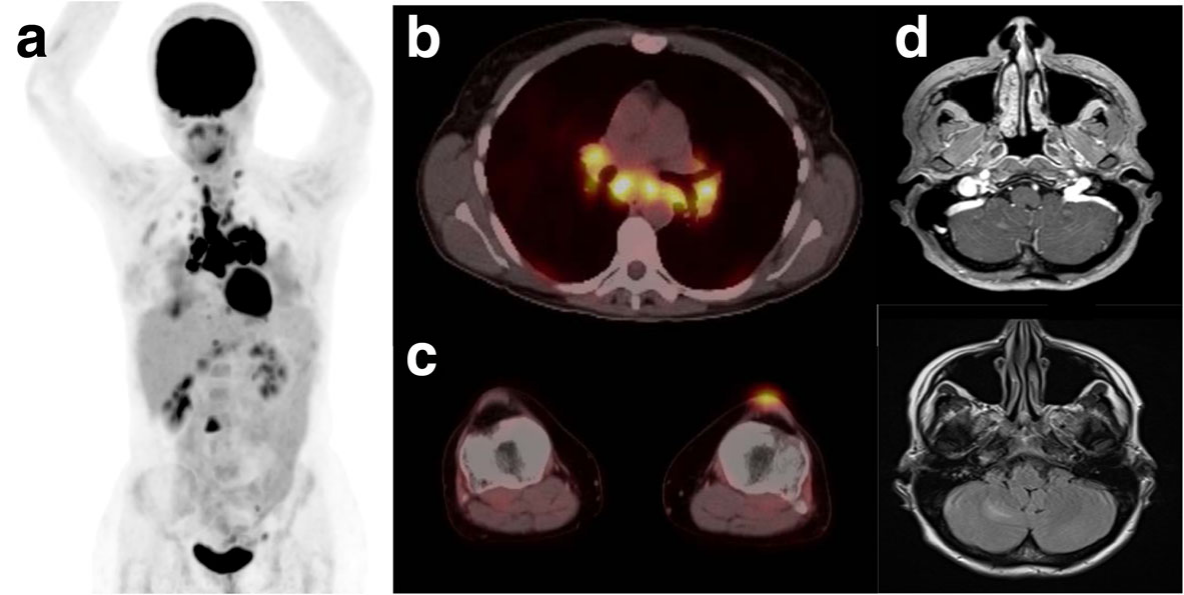
PET-CT scan after Nivolumab + Ipilimumab Induction and on Maintenance Nivolumab. **a** Scout PET scan showing cervical/mediastinal/inguinal adenopathy and vertebral lesions. **b** Cross section of mediastinum with increased uptake in mediastinal nodes (SUV_max_ 16 in paratracheal node). **c** Cross section of knees with increased uptake in left soft tissue nodule (SUV_max_ 2.7). **d** MRI brain with interval improvement of right cerebellar metastases

Prior to cycle 1 of maintenance nivolumab, she developed a scaly lesion of left pretibial skin and underlying subcutis, which grew after her first treatment. Fine needle aspiration (FNA) of the lesion revealed non-caseating granulomatous inflammation without evidence of malignancy. Given the proximity to her primary lesion and history of subcutaneous melanoma metastases, a PET-CT scan was performed. The left pretibial lesion was FDG-avid, and the scans also revealed new supraclavicular, mediastinal, right hilar and left iliac adenopathy, as well as subcutaneous left pretibial and right calf nodules (Fig. 3). The baseline subcarinal and left hilar lymphadenopathy had increased in size, but the hepatic, soft tissue, and brain lesions had resolved (Fig. 3).

**Fig. 4:**
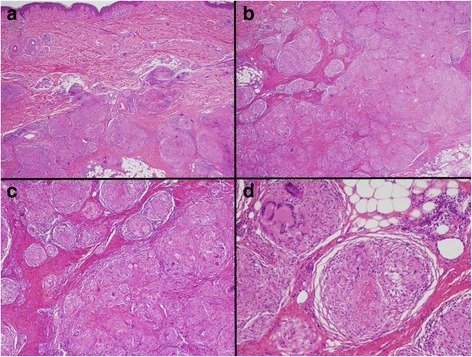
Hematoxylin and Eosin Staining of Pre-Tibial Soft Tissue Nodule. Microscopic hematoxylin and eosin (H&E) section showing (A) large, deep dermal collections of non-caseating granulomas (**a**, 2×) extending into the subcutaneous tissue (**b**, 2×). Higher magnification shows tightly formed granulomas containing multinucleated giant cells separated by fibrous connective tissue (**c**, 4×). Scattered mature appearing lymphocytes are seen surrounding the granulomatous inflammation (**d**, 10×)

**Fig. 5:**
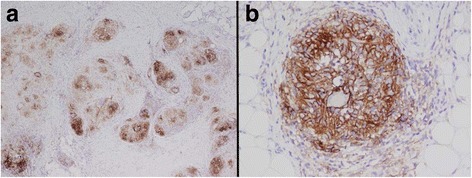
PD-L1 Staining of Pre-Tibial Soft Tissue Nodule. PD-L1 antibody stained section of the granulomatous inflammation shows strong membranous staining of the histiocytes within the granulomas and scattered positive lymphocytes (**a**, 4×; **b**, 20×)

Given the mixed clinical and radiographic results, excisional biopsy of the left pretibial lesion was performed and revealed non-necrotizing granulomatous inflammation consistent with tumefactive sarcoidal granulomas (Fig. 4). In addition, endobronchial ultrasound with FNA of 11R hilar and station 7 subcarinal lymph nodes both demonstrated granulomatous inflammation without evidence of malignancy. AFB, bacterial, and fungal cultures showed no growth. In further support of a diagnosis of sarcoidosis, angiotensin converting enzyme (ACE) level was elevated to 73 U/L (normal 8–52 U/L). To obtain preliminary information on the mechanism for growth of sarcoid lesions in the setting of PD-1 blockade, the skin biopsy tissue was stained for PD-L1 expression, and demonstrated strong PD-L1 staining of the histiocytes and lymphocytes within the granulomas (Fig. 5).

Throughout this evaluation, she remained asymptomatic with normal electrocardiogram and pulmonary function testing. Initially, the decision was made to hold immunotherapy without instituting steroids. After 6 weeks, no new clinical lesions or symptoms appeared and there were no changes in size or extent of the lymphadenopathy. With discussion of the risks and benefits of further treatment, she preferred to resume therapy with single agent nivolumab. At the time of this report, she remains on nivolumab monotherapy with good tolerance and melanoma control. The thoracic lymphadenopathy is stable and no new lesions or clinical symptoms have developed.

## Conclusions

The diagnosis of sarcoidosis in melanoma patients has been made in a diverse array of clinical settings, including sequential and/or concurrent diagnoses, and following initiation of anti-melanoma therapy. To date, sarcoidosis development has been reported in untreated melanoma patients and those managed with interferon, peptide vaccine, vemurafenib, ipilimumab and nivolumab [5–14, 16–21]. To our knowledge, this is the first reported incident of sarcoidosis with combination ipilimumab and nivolumab immunotherapy.

Recent data suggest an incidence of sarcoidosis in melanoma patients of roughly 0.58%, which is substantially higher than the 0.005% incidence of the general population [16]. Our patient had mediastinal and left hilar lymphadenopathy on baseline scans with FDG-uptake comparable with other lesions. The mediastinal and hilar lymph nodes did not respond to immunotherapy treatment, despite response in all other sites present on baseline evaluation, and the adenopathy progressed with the development of the biopsy-proven cutaneous sarcoid lesions. FNA of the subcarinal lymph node present prior to treatment demonstrated granulomatous inflammation, raising the likelihood that the sarcoid process had already started prior to initiation of immunotherapy.

The clinical manifestations of sarcoidosis are diverse, most commonly with pulmonary and cutaneous findings, and are patient-specific with no pathognomonic pattern [16]. As the clinical findings can vary greatly and can occur long after immune therapy is completed, clinicians must be aware of the possibility of granuloma formation that mimics metastatic disease on imaging and examination. Tissue remains the gold standard for diagnosis.

Sarcoidosis is a multi-system granulomatous disorder of unclear etiology characterized by the presence of CD4+ Th1-like cells that initiate granuloma formation in various tissues [22]. These CD4+ Th1 cells secrete predominantly interleukin-2 and interferon-γ to enhance macrophage TNF-α production which results in a compartmentalized accumulation of multinucleated giant cells and epithelioid cells surrounded by CD8+ T-cells, regulatory T-cells, and fibroblasts [22, 23]. Th17 cells are a subset of CD4+ effector T cells that produce inteleukin-17 and are linked with tissue inflammation and autoimmunity [23–25]. Von Euw et al. demonstrated that treatment with the anti-CTLA4 antibody tremelimumab resulted in an increase in Th17 CD4+ cells in peripheral blood [25]. Patients with irAEs from tremelimumab therapy showed an increase in Th17 cells in their post-treatment blood assessments. In a potential link with anti-CTLA4 therapy and sarcoidosis development, bronchoalveolar lavage samples and lung biopsy specimens from patients with sarcoidosis also contain high numbers of Th17 effector CD4+ cells [23, 24].

PD-L1 staining on the sarcoid tissue from our patient was diffusely positive. In 2014, Braun et al. demonstrated increased expression of PD-1 on CD4+ T cells in both peripheral blood and BAL fluid samples from patients with sarcoidosis [26]. There was also upregulation of PD-L1 in sarcoid granulomas. The authors further showed that blockade of the PD-1 pathway, in vitro, restored the proliferative capacity of sarcoid CD4+ cells to the normal range, a pattern seen in spontaneous clinical resolution of sarcoidosis. Consistent with this, Xu et al. also demonstrated diffuse, strong, membranous positivity for PD-L1 in 100% and PD-L2 positivity in 86% of evaluated sarcoidosis cases [27]. These results suggest a possible therapeutic role for anti-PD-1 therapy in patients with sarcoidosis, though this idea is countered by the cases of sarcoid development in the setting of nivolumab and pembrolizumab therapy. Additional research into these questions will be necessary to fully understand the relative role of these therapeutic agents in the cause and management of sarcoidosis.

In our case, there were multiple variables contributing to the clinical scenario and the driver for the sarcoid progression was unclear. She had received both ipilimumab and nivolumab in combination around the time of the clinical change, but in retrospect also likely had sarcoidosis at baseline. We opted for a break in treatment to allow a period of observation without further immunotherapy or initiation of steroids. In 2016, Johnson et al. detailed the use of ipilimumab in a series of 30 patients with pre-existing autoimmune disease, including two patients with underlying sarcoidosis [28]. One of these patients experienced a sarcoid-exacerbation with hypercalcemia and renal insufficiency managed with steroids and the other patient developed an ocular irAE managed with topical steroids. Our patient had no clinical or radiographic change on observation and after discussion of the risks and benefits we elected to continue with nivolumab. To date, there has been neither change in her lymphadenopathy nor the development of any evidence of progression of sarcoidosis or melanoma. With routine use of combination and sequential anti-CTLA-4 and anti-PD1 therapy in melanoma patients, this clinical scenario will likely be encountered with more frequency and providers will be challenged to decide on appropriate anti-melanoma therapy.

In our patient with known metastatic melanoma, had biopsies not been performed the growth of sarcoid would have erroneously led to the conclusion that disease had progressed, which may have resulted in premature cessation of therapy. The differential for new mediastinal adenopathy and cutaneous lesions in melanoma patients on combination ipilimumab/nivolumab immunotherapy should include sarcoidosis. Biopsy for evaluation of new lesions developing on immunotherapy, particularly in the setting of mixed responses, is favored to help guide decision making. Continued anti-PD1 therapy can be considered for appropriate patients with close observation.

## Abbreviations

ACE: Angiotensin-converting enzyme
AFB: Acid-fast bacilli
BAL: Bronchoalveolar lavage
CD: Cluster of differentiation
CT: Computed tomography
CTLA-4: Cytotoxic T-lymphocyte-associated antigen-4
FDG: Fluorodeoxyglucose
FNA: Fine needle aspiration
IgG4: Immunoglobulin G4
irAEs: Immune-related adverse events
mg/kg: Milligram per kilogram
MRI: Magnetic resonance imaging
PD1: Programmed-death 1
PD-L1: Programmed death-ligand 1
PET: Positron emission tomography
Th: T-helper cell
TNF: Tumor necrosis factor
U/L: Units per liter
